# Assessment of a multiplex PCR and Nanopore-based method for dengue virus sequencing in Indonesia

**DOI:** 10.1186/s12985-020-1294-6

**Published:** 2020-02-13

**Authors:** Samuel C. B. Stubbs, Barbara A. Blacklaws, Benediktus Yohan, Frilasita A. Yudhaputri, Rahma F. Hayati, Brian Schwem, Edsel M. Salvaña, Raul V. Destura, James S. Lester, Khin S. Myint, R. Tedjo Sasmono, Simon D. W. Frost

**Affiliations:** 1 Department of Veterinary Medicine, University of Cambridge, Madingley Road, Cambridge, UK; 2 Eijkman Institute for Molecular Biology, Jakarta, Indonesia; 3 University of Philippines Manila, Manila, Philippines; 4 The Alan Turing Institution, London, UK; 5 Present address: London School of Hygiene and Tropical Medicine, London, UK; 6 Microsoft Healthcare, Redmond, USA

**Keywords:** Dengue virus, Disease surveillance, Virus sequencing, Nanopore sequencing

## Abstract

**Background:**

Dengue virus (DENV) infects hundreds of thousands of people annually in Indonesia. However, DENV sequence data from the country are limited, as samples from outbreaks must be shipped across long-distances to suitably equipped laboratories to be sequenced. This approach is time-consuming, expensive, and frequently results in failure due to low viral load or degradation of the RNA genome.

**Methods:**

We evaluated a method designed to address this challenge, using the ‘Primal Scheme’ multiplex PCR tiling approach to rapidly generate short, overlapping amplicons covering the complete DENV coding-region, and sequencing the amplicons on the portable Nanopore MinION device. The resulting sequence data was assessed in terms of genome coverage, consensus sequence accuracy and by phylogenetic analysis.

**Results:**

The multiplex approach proved capable of producing near complete coding-region coverage from all samples tested ($\overline{x}$ = 99.96%, *n* = 18), 61% of which could not be fully amplified using the current, long-amplicon PCR, approach. Nanopore-generated consensus sequences were found to be between 99.17–99.92% identical to those produced by high-coverage Illumina sequencing. Consensus accuracy could be improved by masking regions below 20X coverage depth (99.69–99.92%). However, coding-region coverage was reduced at this depth ($\overline{x}$ = 93.48%). Nanopore and Illumina consensus sequences generated from the same samples formed monophyletic clades on phylogenetic analysis, and Indonesian consensus sequences accurately clustered by geographical origin.

**Conclusion:**

The multiplex, short-amplicon approach proved superior for amplifying DENV genomes from clinical samples, particularly when the virus was present at low concentrations. The accuracy of Nanopore-generated consensus sequences from these amplicons was sufficient for identifying the geographic origin of the samples, demonstrating that the approach can be a useful tool for identifying and monitoring DENV clades circulating in low-resource settings across Indonesia. However, the inaccuracies in Nanopore-generated consensus sequences mean that the approach may not be appropriate for higher resolution transmission studies, particularly when more accurate sequencing technologies are available.

## Background

Dengue virus (DENV) is a mosquito-borne pathogen in the genus *Flavivirus,* family *Flaviviridae*. The virus is predominantly found circulating in urban or semi-urban areas of tropical regions where its vectors, *Aedes aegypti* and *Aedes albopictus,* are present [1] and is estimated to infect 390 million people each year, approximately 100 million of whom exhibit clinical signs [2].

In Indonesia, where all four serotypes of DENV co-circulate, an average of 130,000 dengue virus cases were reported annually between 2004 and 2010 [3]. This was the highest case rate in Asia, and the second highest worldwide. However, genetic information for Indonesian isolates of DENV is limited. At present, there are 142 complete coding region sequences from Indonesia deposited in the GenBank sequence database (www.ncbi.nlm.nih.gov/genbank), only 31 of which are derived from samples taken within the past 10 years (i.e. since 2009). Availability of such sequencing data can be a vital tool in studying the epidemiology of DENV, as it can be used to improve our understanding of transmission routes, aid early detection of new outbreaks and epidemics, and support the public health response [4].

DENV sequencing is made complicated for large, developing countries such as Indonesia, as sequencing facilities may not be immediately available at the site of an outbreak. Instead, samples from suspected DENV cases are often stored in sub-optimal conditions for long periods of time, particularly when the location of the outbreak is remote. These samples must then be transported long distances to be sequenced in the few laboratories equipped to do so. Unsurprisingly, many such samples arrive degraded, rendering sequencing difficult.

Historically, molecular phylogenetic studies of DENV have relied on sequencing the envelope (*E*) gene (~ 1.5 kbp) (e.g [5]), which encodes the major surface antigen of the virus [6]. However greater phylogenetic resolution can be achieved by considering a larger sequence, such as the complete (~ 10.2 kbp) coding region of the virus. The existing method for achieving this involves serotype-specific PCR amplification of 5 long (1–2.5 kbp) amplicons in single-plex reactions, followed by gel electrophoresis for band purification, and sequencing on the Sanger capillary or Illumina platforms [7].

In the above method, PCR amplification of the DENV genome prior to sequencing serves to increase the sensitivity of the approach, facilitating recovery of sequence data from clinical samples with low viral loads. Enrichment of the target of interest also reduces the amount of sequence data required to generate sufficient coverage of each genome, enabling a greater number of samples to be sequenced in multiplex. This allows for savings in both sequencing costs and time, which are particularly vital factors for low-resource settings and outbreak situations respectively. However, it is important to note that PCR amplification can fail due to discordance between the primers and viral genome, resulting in loss of sequencing coverage. Such failure is most likely when attempting to amplify divergent strains of a virus, such as newly emerging strains, or those from disparate regions upon which the primers were not designed.

An alternative approach known as metagenomics involves sequencing all nucleic acid present in a sample without the use of a targeted PCR step. This has been performed for DENV sequencing on several platforms including the ONT MinION device [8]. The major benefit of the metagenomic approach is its broad specificity. It is theoretically capable of producing sequence data from any infecting virus, regardless of serotype or strain, without need for a priori identification. However, such an approach can lack sensitivity, as viral genetic material is generally present in very small amounts compared to that of the host, particularly when viral load is low. The poor sensitivity of metagenomic sequencing therefore necessitates a reduction in the number of samples that can be sequenced simultaneously, greatly increasing costs and making the approach unsuitable for low-resource settings.

A third possible approach to DENV sequencing involves the use of oligonucleotide probes bound to biotinylated magnetic beads [9]. These probes are used to specifically bind and separate viral nucleic acid, and are therefore restricted by their ability to anneal to the viral genome, similarly to PCR primers. However, in contrast to PCR, variation between viral strains can be accounted for by employing many probes in a single reaction without interference, allowing for a broader specificity. Nonetheless, this method is slow due to the inefficient recovery of nucleic acid, and the specialised manufacture of the bead-bound probes is expensive in comparison to PCR primers.

Prioritising the importance of affordability and ease of implementation, we selected a PCR-based method for amplifying and sequencing the complete coding region of DENV clinical isolates, with the aim of developing it for use in resource-limited settings across Indonesia. The method makes use of the ‘Primal Scheme’ multiplex PCR-tiling approach originally developed for field sequencing of two other arboviruses: Zika virus and chikungunya virus [10], and has previously been used to detect an isolate of cosmopolitan genotype DENV-2 in Angola [11]. An important aspect of this approach is that the amplicons produced are short (400 bp), making it better suited to low quality samples compared to the conventional, long-amplicon, single-plex approach in use at present. We tested the approach by sequencing the amplicons on the relatively affordable and highly portable Oxford Nanopore Technologies (ONT) MinION sequencing device, which can be used in smaller, poorly-equipped laboratories, or even taken to the sampling site. We performed amplification and sequencing on a range of clinical samples and reference material to assess genome coverage, and compared this to results generated using the current single-plex approach. Several of the samples were also sequenced in parallel on the Illumina MiSeq in order to compare the platforms in terms of consensus sequence accuracy.

## Methods

### Design of multiplex primer sets

The ‘Primal Scheme’ primer design tool (primal.zibraproject.org) was used to design multiplex primer sets that would produce multiple 400 bp amplicons covering the entire coding region of DENV, with a 50 bp overlap between adjacent amplicons to facilitate assembly of the sequence data. Primer design was based on all available full genome DENV sequences from Indonesia obtained from NCBI GenBank (www.ncbi.nlm.nih.gov/genbank). The full list primer sequences used for this study can be found in Additional file 1. As the primer sets are expected to undergo refinement, the current version (v1) and future versions have also been made available at: doi.org/10.5281/zenodo.3516727.

### Samples

Two sets of DENV positive clinical samples were used in this study; 10 archived serum samples from patients in Indonesia, and 4 serum samples from patients in The Philippines.

The method was also tested using the AmpliRun dengue RNA control set (VirCell, Granada, Spain), consisting of purified RNA from each of the four DENV serotypes. These control samples ranged in concentration from 12,500–20,000 viral genome copies / μl. Their complete genome sequences are available in GenBank: DENV-1 (KM204119.1), DENV-2 (KM204118.1), DENV-3 (KU050695.1), DENV-4 (KR011349.2), however they were re-sequenced for the purposes of this study to account for any mutations introduced through passaging of the source material.

### RNA extraction, reverse transcription and PCR amplification

RNA was extracted from 200 μl of serum using the MagNA-Pure LC Total Nucleic Acid kit (Roche) and eluted in 50 μl of elution buffer. 7 μl of extracted RNA were reverse transcribed in a 20 μL reaction, using 50 ng of random hexamers (Invitrogen) and Superscript III RT enzyme (Thermo Fisher Scientific), as per the manufacturer’s instructions. Second-strand synthesis of cDNA was performed by adding 2.5 U of large Klenow fragment (New England Biolabs, NEB) to each reaction and incubating at 37 °C for 60 min followed by 75 °C for 10 min.

Two multiplex PCR reactions were performed per sample, with the primers separated into two pools (P1 and P2) to avoid interference between overlapping amplicons (see S1 Primer List for pooling information). PCR reactions contained 2 μL of cDNA reaction as input, 0.25 μL of Q5 polymerase (NEB), 5 μL of Q5 HF reaction buffer, 0.5 μL of 10 mM dNTPs and a variable volume of primer pool 1 or 2 (10 μM) to a final concentration of 0.015 μM per primer (e.g. 1.5 μL for DENV-1, which contains 40 primers per pool). RNase-free water was added to a final volume of 25 μL. A two-step PCR amplification was performed as follows: 98 °C for 30 s, followed by 40 cycles of 98 °C for 15 s and 65 °C for 5 min. Following amplification, the two reaction pools were combined into 50 μL and amplicons were purified using an equal volume of AMPure beads (Agencourt), eluting in 20 μL of RNase-free water.

Five single-plex PCR reactions were also performed for each sample using 2 μl of the cDNA reaction as input. Long (1–2.5 kbp) amplicons were produced using serotype-specific primers following the protocols described by Ong et al. [12] (DENV1–3) and Sasmono et al. [13] (DENV-4), using *Pfu* Turbo DNA Polymerase reagents (Thermo Fisher Scientific) in accordance with the manufacturer’s instructions. Amplicons were purified by electrophoresis on a 1% agarose gel and extracted using the Monarch Gel Purification Kit (NEB).

### Nanopore sequencing and consensus sequence generation

Eighty ng of purified amplicons from each sample were barcoded using the Oxford Nanopore Technologies (ONT) Native Barcode kit (EXP-NBD103 or EXP-NBD104). Following barcoding, amplicons were pooled in equal volumes and 80 ng was used as input for the ONT 1D Ligation Sequencing kit (SQK-LSK108 or SQK-LSK109) following ONT’s 1D Native Barcoding Genomic DNA protocol (available from https://community.nanoporetech.com/protocols).

Libraries produced by the RNA standards and the Philippines samples were sequenced on the ONT MinION using R.9.4 flow-cells and MinKNOW software v1.11.5 (ONT). The ten Indonesian samples were sequenced at a later date using R.9.4 flow-cells and MinKNOW software v3.1.4.

Raw FAST5 files were base-called using Guppy v3.1.5 (ONT) and reads with a q-score below 7 were discarded. Run statistics are shown in Table 1. Qcat v1.0.7 (ONT) was used with default settings for demultiplexing and the de-multiplexed FASTQ files were aligned to the serotype-appropriate dengue reference sequence (NC_001477.1 for DENV-1, NC_001474.2 for DENV-2, NC_001475.2 for DENV-3, and NC_002640.1 for DENV-4) using BWA mem v0.7.17 (option -x ont2d) [14].

**Table 1:** Overview of MinION run statistics for samples in this study

Sample Origin	Library Kit	MinKNOW version	Run Length (hours)	Reads Generated	Mean Q-score	Reads > Q7 (%)	Reads > Q10 (%)
RNA Standards	SQK-LSK108	1.11.5	14	353,266	6.6	51.1	0.1
Philippines	SQK-LSK108	1.11.5	20	147,281	7.4	78.0	0.0
Indonesia	SQK-LSK109	3.1.4	14	1,190,228	8.6	86.4	11.7
Indonesia	SQK-LSK109	3.1.4	6	2,185,287	8.8	89.6	12.3
Indonesia	SQK-LSK109	3.1.4	5	3,317,009	9.0	92.0	16.8

The resulting BAM alignment files were sorted and indexed using Samtools v1.7 [15] and primer sequences were soft clipped using BamCLIPPER [16] (options -u 50 -d 50). Consensus sequences were called from the primer clipped BAM files using bcftools v1.9 [15] (options -c -v) and a simple pileup method (options -Q 7 -d 1000), masking regions below 1x and 20x coverage depth. The base-called FASTQ reads were realigned to the resulting draft consensus sequences, and primer sequences were again clipped. Consensus correction was performed using Nanopolish variants v 0.11.1 [17] (options --ploidy 1 --min-flanking-sequence 10). Variant calls were filtered to include only those with a quality score of > 200 and a support fraction > 70% before being applied to generate the final consensus genomes.

### Illumina library sequencing and consensus sequence generation

Illumina sequencing was also performed on amplicons from both the RNA control samples and the clinical samples from the Philippines. Amplicons generated by the single-plex PCR approach were purified by agarose gel electrophoresis, quantified using the Qubit high sensitivity double-stranded DNA kit (Thermo Fisher Scientific) and pooled in equimolar amounts. 50 ng of input DNA from each sample was fragmented to an average of 400 bp by sonication (Covaris E220). Barcoded Illumina multiplex libraries were prepared from the fragmented DNA using NEBNext multiplex oligos and the NEBNext Ultra II kit as per the manufacturer’s instructions (NEB). The resulting libraries were sequenced on the Illumina MiSeq using v2 chemistry producing an average of 1,064,176,250 bp paired-end reads per sample (range: 924,256 – 1,264,182 reads).

Demultiplexed, paired-end FASTQ files were imported into CLC Genomics Workbench v7.5.1 (Qiagen) for analysis. The CLC trimming algorithm was used with default settings to remove primer sequences and discard low-quality reads resulting in an average of 969,613 reads remaining (range: 838,897 – 1,147,122 reads). Trimmed reads were mapped to the serotype-appropriate dengue virus RefSeq sequence, requiring a minimum of 64% identity (80% similarity over 80% of the read length), achieving an average coverage depth of > 15,000X for all samples. Consensus sequences were called from the resulting alignments requiring a minimum of 1x coverage depth, and a minimum support fraction of 20% for calling ambiguities.

### Assessment of sequence accuracy

Nucleotide sequence similarity between the Nanopore-generated consensus sequences and the Illumina-generated sequences was assessed by pairwise alignment using EMBOSS Needle [18].

### Phylogenetic analysis

Reference sequences were extracted from the ‘Online Dengue Virus Typing Tool’ (available at www.krisp.org.za/tools.php) and additional sequences from Indonesia added for clarity. The sequences were trimmed to the beginning and end positions of the single open reading frame. Multiple sequence alignments of the DENV coding region were generated using MUSCLE v 3.8.1551 [19]. Phylogenetic trees were reconstructed using maximum likelihood in IQTREE v 1.6.11 [20], using model selection [21], a more thorough nearest neighbour interchange search (‘-allnni’), and 1000 iterations of the ultrafast bootstrap approximation (−bb 1000) [22]. The resulting trees were visualised using ggtree [23] and distances between taxa were calculated using the cophenetic.Phylo function of the ape package v 5.3 [24] in R v 3.5.1 [25].

## Results

### Testing the multiplex PCR and Nanopore sequencing on RNA reference material

Amplicons were generated from the four DENV RNA samples using the multiplex PCR approach and sequenced on the Nanopore MinION. The sequencing run generated 8604–16,654 reads per sample passing the quality filters (Q-score ≥ 7) (Table 1).

Alignment of the reads to the appropriate RefSeq genome demonstrated that full coverage of the coding region was achieved for all four serotypes (Table 2). The resulting consensus sequences were on average 99.49% identical to the Illumina-generated consensus sequences used as references.

**Table 2:** Nanopore sequencing metrics for DENV RNA standards, using the multiplex (400 bp amplicon) approach

Serotype	Genotype	Reads passing QC	Coding-Region Coverage (%)		Nucleotide Consensus Similarity (%)	
			1X	20X	1X	20X
1	I	8604	100	96.51	99.92	99.88
2	IV	14,265	100	87.56	99.50	99.81
3	V	8337	100	89.93	99.28	99.69
4	I	16,654	100	89.96	99.25	99.72

**Fig. 1:**
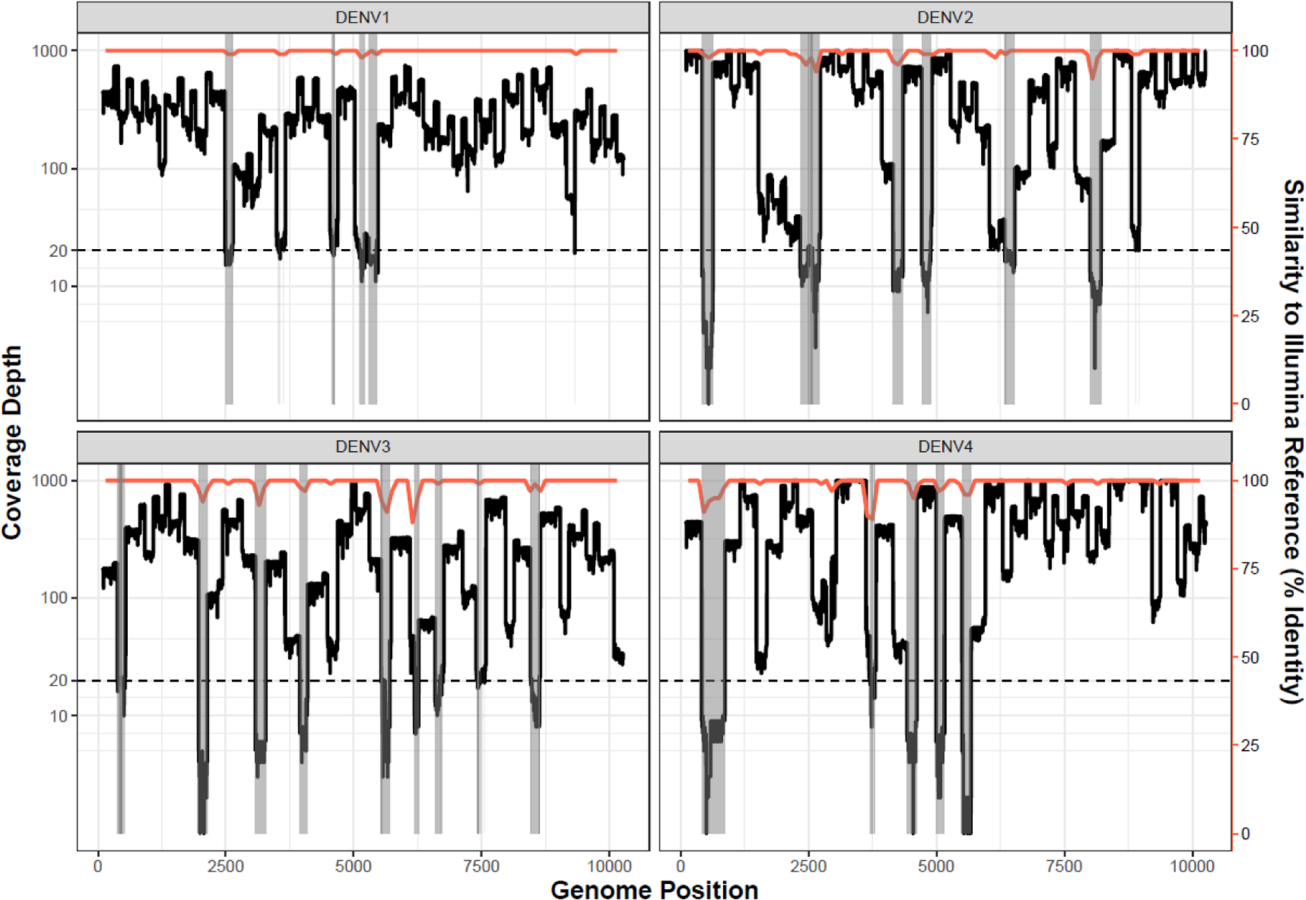
Nanopore sequencing coverage for DENV control RNA samples, using the multiplex (400 bp amplicon) approach. Nanopore sequencing coverage depth for dengue virus control RNA samples, using the multiplex PCR approach is plotted in black against the left-hand y-axis, with the read depth threshold of 20X indicated by the dotted line. Coverage depth is capped at 1000X. The nucleotide similarity of the Nanopore-generated sequence to the Illumina-generated reference sequence is shown in red against the right-hand y-axis. The shaded areas identify regions where the coverage depth fell below the low coverage threshold of 20X, to allow comparison with the similarity plot

However, sequencing coverage depth was uneven, with only 87.56–96.51% of the DENV1–4 genomes covered by 20 or more reads. These regions of low coverage coincided with drops in consensus sequence accuracy (Fig. 1). Masking regions of the genome with less than 20X coverage depth improved the overall accuracy of the consensus sequence in 3/4 cases, increasing the average consensus identity to 99.78%. However, masking these regions also resulted in a loss of genome coverage ($\overline{x}$ = 9.01%) (Table 2).

The Nanopore MinION was also used to sequence amplicons generated using the single-plex PCR approach. The resulting reads generated consensus sequences 99.93–99.99% identical to the Illumina references ($\overline{x}$ = 99.97%), containing an average of 4 mismatches (range = 1–7) (Table 3). These consensus sequences were on average 0.48% more accurate than their multiplex-generated counterparts.

**Table 3:** Nanopore sequencing metrics for DENV RNA standards, using the single-plex (2500 bp amplicon) approach

Serotype	Genotype	Reads Passing QC	20X Coding-Region Coverage (%)	Nucleotide Consensus Similarity (%)
1	I	31,601	100	99.99
2	IV	8274	100	99.93
3	V	14,300	100	99.98
4	I	31,486	100	99.96

### Testing on Indonesian clinical samples

The multiplex PCR approach was next tested on a set of clinical samples from Indonesia (*n* = 10). These samples included representatives of each of the four DENV serotypes across a range of viral loads (Ct values 15.2–37.9). All of the samples produced PCR products of the expected size (~ 400 bp) and the resulting amplicons were sequenced on the Nanopore MinION, producing 33,908–82,891 reads ($\overline{x}=\mathrm{57,948}\Big)$.

**Fig. 2:**
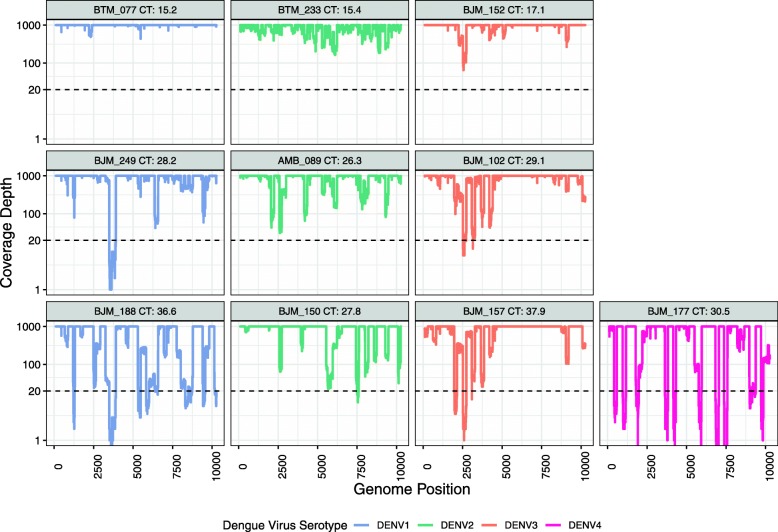
Nanopore Sequencing Coverage of Indonesian Clinical Isolates using the Multiplex PCR Approach. The multiplex PCR approach was used to amplify DENV1–4 from 10 clinical samples from Indonesia. These samples were selected to cover a range of viral loads, as estimated by Ct values from the diagnostic qRT-PCR. The resulting amplicons were sequenced on the Nanopore MinION. Coverage depth for each sample is plotted, with the read depth threshold of 20X indicated by the dotted line. Coverage depth is capped at 1000X

The average coding-region coverage at 1X read depth was 99.80% across the 10 samples (Table 4), and complete coverage was achieved for 8 of the 10. At 20X read depth, average coverage fell to 95.84%. Drops in coverage below 20X were more frequent in samples with higher Ct values (Fig. 2). Those with a Ct value of 25 or less (*n* = 3) generated an average of 100% coverage. The average coverage fell to 98.55% for samples with a Ct value between 25 and 30 (*n* = 4), and was further reduced to 88.06% for those with Ct values greater than 30 (*n* = 3).

**Table 4:** Sequencing coverage of clinical isolates from Indonesia, using the multiplex and single-plex amplification approaches

ID	Serotype	Genotype	Ct value	Reads passing QC	Coding-Region Coverage (%)		Single-plex Amplification Result (5′ → 3′)				
					1X	20X	1	2	3	4	5
BTM-077	1	I	15.2	76,301	100	100	**+**	**+**	**–**	**+**	**+**
BJM-249	1	I	28.2	51,554	100	95.89	**+**	**–**	**–**	**+**	**–**
BJM-188	1	I	36.6	57,002	99.95	86.58	**–**	**–**	**–**	**+**	**–**
BTM-233	2	Cosmopolitan	15.4	33,908	100	100	**+**	**+**	**+**	**+**	**+**
AMB-089	2	Cosmopolitan	26.3	42,694	100	100	**+**	**+**	**+**	**+**	**–**
BJM-150	2	Cosmopolitan	27.8	82,891	100	99.27	**+**	**–**	**–**	**–**	**–**
BJM-152	3	I	17.1	67,280	100	100	**+**	**+**	**+**	**+**	**+**
BJM-102	3	I	29.1	50,803	100	99.05	**+**	**+**	**–**	**+**	**–**
BJM-157	3	I	37.9	71,226	100	96.22	**–**	**–**	**–**	**–**	**–**
BJM-177	4	II	30.5	45,823	98.02	81.38	**–**	**–**	**+**	**–**	**–**

The same 10 samples were also amplified using the single-plex PCR approach as a comparison (Table 4). On average, the approach only produced 2.6 of the 5 amplicons required to cover the DENV coding-region. As with the multiplex approach, samples with the lowest Ct values (< 20) were the most successful, producing 93% of amplicons (14/15), whereas those with Ct values greater than 25 produced only 34% (12/35).

### Testing on non-Indonesian clinical samples

The multiplex method was next applied to four clinical samples from DENV-infected patients from The Philippines, in order to test how the method performed when working with viral strains from countries outside of Indonesia. All of the samples produced PCR products of the expected size (~ 400 bp) and the resulting amplicons were sequenced on the Nanopore MinION, producing 6852–12,972 reads ($\overline{x}=\mathrm{8,048}\Big)$.

**Fig. 3:**
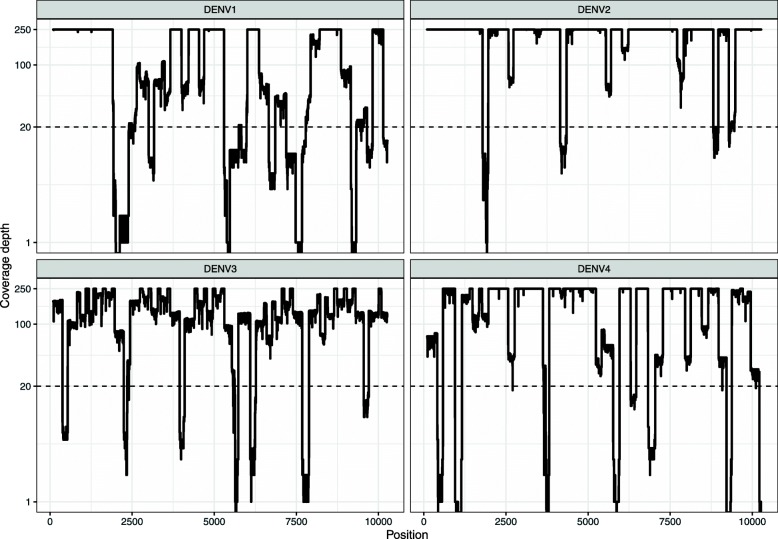
Nanopore Sequencing Coverage of Pilipino Clinical Isolates using the Multiplex PCR Approach. The multiplex PCR approach was used to amplify DENV1–4 from 4 clinical samples from The Philippines. The resulting amplicons were sequenced on the Nanopore MinION. Coverage depth for each sample is plotted, with the read depth threshold of 20X indicated by the dotted line. Coverage depth is capped at 250X

The average coding-region coverage at 1X read depth was 99.90% across the 4 samples (Table 5). At 20X read depth, average coverage fell to 88.40%. DENV-1 produced several particularly large drops in coverage depth compared to the other isolates (Fig. 3), resulting in only 79.22% of the coding-region being covered at 20X read depth. Consensus sequences were again generated and compared to Illumina-generated reference sequences by pairwise-nucleotide alignment (Table 5). Consensus sequences produced using all regions covered by 1 or more read were found to be 99.17–99.80% identical to the Illumina-generated sequence ($\overline{x}=99.45\%\Big)$. Masking regions with a read depth below 20X improved consensus sequence accuracies to 99.70–99.92% ($\overline{x}=99.80\%\Big)$, at the expense of coverage.

**Table 5:** Sequencing metrics for clinical isolates from The Philippines

ID	Serotype	Genotype	Reads passing QC	Coding-Region Coverage (%)		Nucleotide Consensus Similarity (%)		Single-plex Amplification Result (5′ → 3′)				
				1X	20X	1X	20X	1	2	3	4	5
130,158	1	IV	6852	99.80	79.22	99.57	99.86	+	+	+	+	–
130,104	2	Cosmo.	12,932	99.99	94.57	99.80	99.92	+	+	+	+	–
1,601,002	3	I	4605	99.93	89.40	99.17	99.70	+	+	+	+	+
130,343	4	II	7805	99.90	90.40	99.24^a^	99.70^a^	+	+	+	+	–

^a^Several of the single-plex reactions were unsuccessful (−), and had to be repeated using primers from the multiplex primer set to generate the reference amplicons that were sequenced on the Illumina platform. The single-plex approach was unable to generate an amplicon covering the 3′ end of the DENV-4 genome, despite repeated attempts with a range of primers. Therefore, it should be noted that the accuracy of the multiplex-generated consensus sequence is not for the full coding region, but only accounts for 10,117 of the 10,163 coding bases

The single-plex PCR approach was again used to produce the amplified product for Illumina sequencing, however 3 of the 4 samples failed to generate one of the expected products (Table 5). Complete coverage of DENV-1 and 2 was therefore achieved by replacing the published primer sets with primers taken from the multiplex set. Following several attempts, only a truncated version of the most 3′ DENV-4 amplicon could be produced, and so the accuracy of the Nanopore-generated consensus could be assessed for 10,117 of the 10,163 coding bases only.

### Assessment of the Nanopore-generated consensus sequences by phylogenetic analysis

**Fig. 4:**
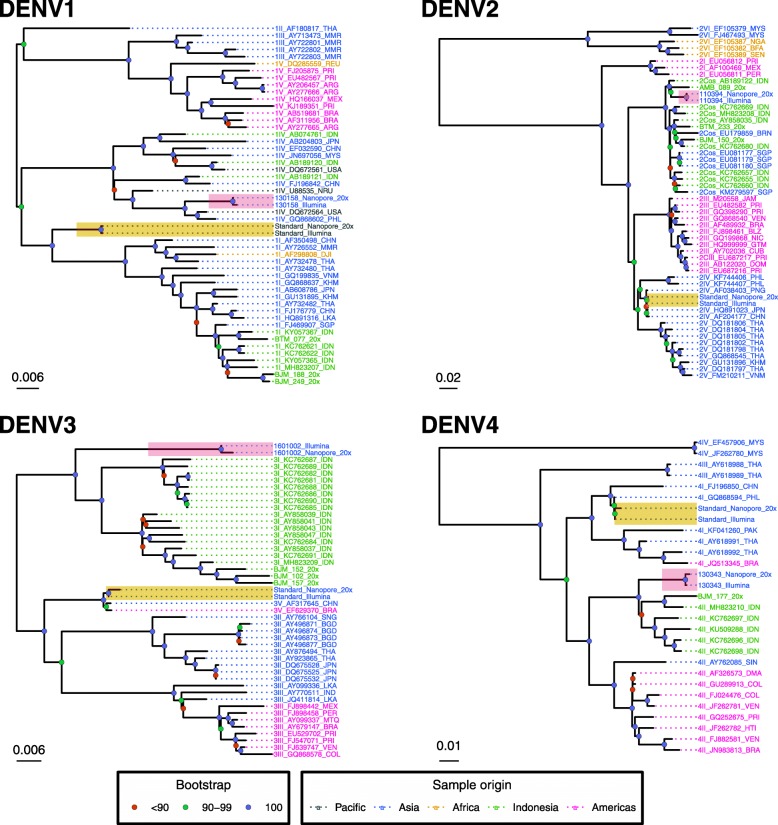
Phylogenetic analysis of Illumina- and Nanopore-generated consensus sequences. Bootstrap phylogenies of complete DENV coding regions were constructed using Nanopore and Illumina consensus sequences and a selection of genotype reference sequences. Nanopore consensus sequences were generated for all samples using the short amplicon approach, with regions below 20X coverage depth masked. Illumina consensus sequences were generated for the RNA standards and Pilipino samples using the long-amplicon approach. Sequence names are coloured to denote geographical origin, and internal nodes of the tree are coloured to demonstrate bootstrap values (blue = 100%, green = 90–99% and red = < 90%). Monophyletic clades formed by the Nanopore and Illumina-generated consensus sequences are highlighted in yellow for the RNA standard samples, and red for the Pilipino clinical samples

**Fig. 5:**
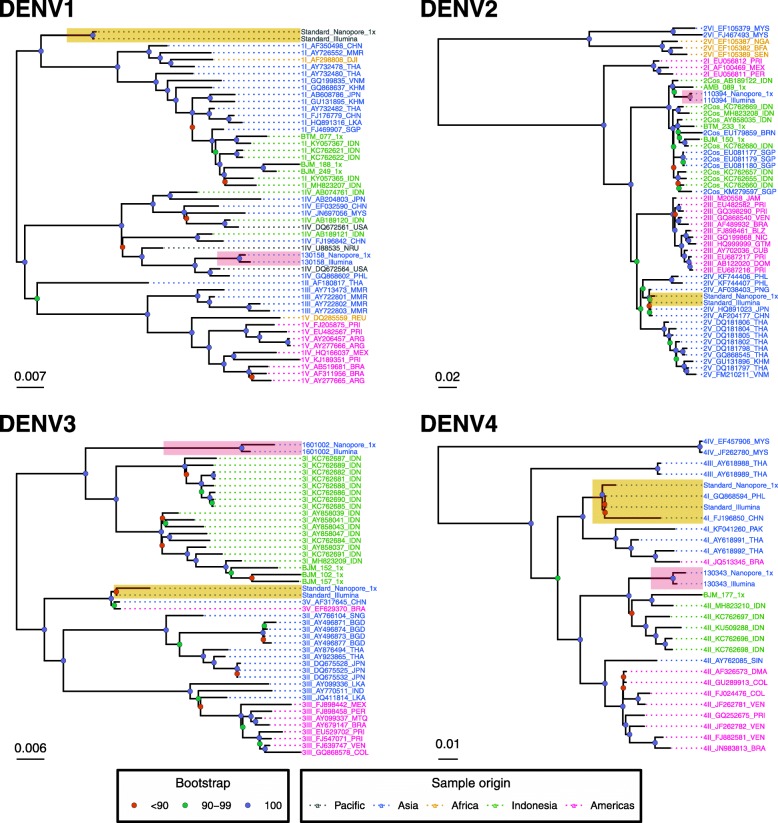
Phylogenetic analysis of Nanopore (1x) and Illumina-generated consensus sequences. Bootstrap phylogenies of complete DENV coding regions were constructed using Nanopore and Illumina consensus sequences and a selection of genotype reference sequences. Nanopore consensus sequences were generated for all samples using the short amplicon approach with only regions below 1X coverage depth masked. Illumina consensus sequences were generated for the RNA standards and Pilipino samples using the long-amplicon approach. Sequence names are coloured to denote geographical origin, and internal nodes of the tree are coloured to demonstrate bootstrap values (blue = 100%, green = 90–99% and red = < 90%). Clades formed by the Nanopore and Illumina-generated consensus sequences are highlighted in yellow for the reference samples, and red for Pilipino samples

Phylogenies were constructed using the Nanopore- and Illumina-generated coding region sequences and a set of reference sequences for each DENV serotype. Separate phylogenies were constructed using Nanopore consensus sequences masked below 20x coverage depth (Fig. 4) and 1x coverage depths (Fig. 5). The Nanopore consensus sequences generated from 20 x depth all formed monophyletic clusters with their Illumina-generated counterparts. The Nanopore-generated sequences for DENV-1-3 at 1x depth also formed monophyletic clades with their Illumina-generated counterparts, however the DENV4 sequence was separated from its Illumina counterpart by GQ868594, a sequence generated from the same viral isolate. Pairwise phylogenetic distance between the Nanopore- and Illumina-generated sequence tips averaged 0.001975 for those generated using regions of >20X coverage, and 0.005685 for those generated using 1X coverage (Table 6).

**Table 6:** Pairwise phylogenetic distance between Nanopore and Illumina-generated consensus sequences

Serotype	Genotype	Source	Genetic Distance from Illumina-generated consensus (1X)	Genetic Distance from Illumina-generated consensus (20X)
1	I	Reference	0.00107	0.00092
1	IV	Clinical	0.00422	0.00123
2	IV	Reference	0.00517	0.00153
2	Cosmopolitan	Clinical	0.00196	0.00051
3	V	Reference	0.00735	0.00277
3	I	Clinical	0.00890	0.00299
4	I	Reference	0.00856	0.00311
4	II	Clinical	0.00825	0.00274

Sequences from the Indonesian samples formed distinct clades containing the majority of reference sequences from Indonesia (green labels). The Indonesian clades for DENV-3 and DENV-4 were exclusively composed of Indonesian sequences, whilst the Indonesian DENV-1 clade also contained one sequence from the neighbouring country of Singapore (FJ469907). The Indonesian DENV-2 clade included sequences from several neighbouring South East Asian countries including Brunei (EU179859), Singapore (EU081177, EU081179, EU081180, KM279597) and the Philippines (110394). The Indonesian consensus sequences also clustered by region whenever multiple samples from the same region of Indonesia were included. DENV-1 and DENV-3 samples from Banjarmasin (BJM) in Central Indonesia were clustered, whilst samples from Batam (BTM) in the West, and Ambon (AMB) in the East, clustered separately.

## Discussion

The ONT MinION sequencing device has the potential to revolutionise infectious disease surveillance, thanks to its portability, affordability and ease of set up. This is particularly true for large, developing countries such as Indonesia, where access to main-stream sequencing platforms such as Illumina, Ion Torrent and PacBio is not readily available outside of the best-equipped laboratories.

At present, the relatively high error rate of individual Nanopore reads means that even the most precise consensus sequences only reach ~ 99.9% in accuracy [26], equivalent to approximately 10 errors across the 10.2 kb DENV coding region. In this study, a level of accuracy comparable to the gold standard of Illumina sequencing was achieved, but this was limited to regions with sufficient depth of coverage (i.e. ≥ 20 X). However, similarity between the Nanopore and Illumina-generated consensus sequences was sufficient for them to form monophyletic clades on phylogenetic analysis. The phylogenetic analysis also demonstrated clear clustering of the Indonesian consensus sequences with reference sequences from the same country, as well as between samples from the same geographical region within Indonesia. This demonstrates that the accuracy of Nanopore sequencing is sufficient for surveillance of DENV in Indonesia, as the principal aim of such research would be to identify the origin of viral clades and so inform public health epidemiology. It is however unclear whether this level of error would be suitable for viral transmission studies where the level of evolutionary divergence is low, such as those investigating outbreaks within a single hospital or over a short time period .

Clinical DENV samples can range in both RNA quality and viral load, making genome recovery unreliable. The multiplex PCR approach was therefore tested on real samples across a range of concentrations, in order to assess the method as a means to provide robust amplification of the DENV genome. Sequencing of the amplicons produced 99.5% or greater coverage at the first attempt across all samples; failing to reach 100% coverage for only 4 out of 18. Two of these four were at low concentration by diagnostic qRT-PCR (Ct values > 30) and two were derived from patients outside of Indonesia, suggesting improvement in the primer sets is required to better capture non-Indonesian isolates. Notably, the coverage achieved by the multiplex method was equal to or outperformed the single-plex PCR approach across all clinical samples tested, demonstrating that it is the optimal approach for dealing with low concentration or low-quality samples in the field.

One drawback to the multiplex approach was the unevenness of coverage, as low coverage regions corresponded with drops in consensus accuracy. Whilst it was possible to produce full consensus sequences of greater than 99% accuracy by including regions of coverage depth as low as 1x, our data suggest that consensus sequences are most accurate when called from regions with a higher depth (e.g. 20x), although this can be at the cost of reduced genome coverage.

Improving sequencing depth over the entire genome is therefore of primary importance for the development of this method. Continued use of the approach in the field will facilitate the identification of regions that routinely lack coverage. This information can therefore be used to develop the primer sets to better capture such regions, with each iterative update being made publicly available online (see Methods section). For example, one noticeable outcome from this data-set was the highly uneven coverage of the DENV-1 genotype IV sample, derived from a patient in the Philippines. The Philippines is a neighbouring country to Indonesia, making Pilipino strain incursions into Indonesia likely. Therefore, the DENV-1 primer sets responsible for these regions of low coverage will require alteration to better capture DENV-1 genotype IV strains in the future.

Coverage depth can also be increased by simply running the MinION for a longer period of time, as flow-cells can be run for up to 48 h before they are exhausted. The sequencing runs in this study lasted a maximum of 20 h in order to conserve the flow-cells for future use. However, the majority of samples generated 100% coverage at 1x depth, implying that improved coverage of all regions would be possible if sequencing were to continue. To this end, the ‘real-time’ nature of the device can also be taken advantage of by running software such as RAMPART in parallel (github.com/artic-network/rampart). This software is capable of assessing the number of reads and depth of coverage in real-time, allowing the user to ensure sufficient coverage has been generated before ending the run.

Additionally, recent improvements in ONT library preparation kits, flow-cell technology, and base-calling algorithms have led to significant advancements in both sequence yield and quality. This was noticeable in the data reported here. The initial two sequencing runs, used to sequence the RNA standards and the samples from the Philippines, produced read yields and qualities noticeably lower than those produced when sequencing the Indonesian samples 12 months later, due to differences in library preparation kits and sequencing software. It is therefore anticipated that the accuracy of consensus sequences generated using this approach will continue to improve as aspects of the technology such as the base calling algorithm [26], and the nanopore itself (e.g. the introduction of a double-read head in flow-cell version R10), are further optimised. Such improvements should also allow the minimum read depth required to generate accurate consensus sequences to be reduced, shortening sequencing run times. At present, the entire procedure, from sample receipt to completion of data analysis, takes approximately 20–30 h depending on sequencing run time, and can therefore be completed on the day of receipt if the situation demands it (e.g. in response to an outbreak). In comparison, the same protocol using the Illumina MiSeq platform can take 2–3 times as long, largely due to the fact that a sequencing run alone takes 39 h to complete (v2 500 cycle kit).

## Conclusions

Overall, the approach described here represents a viable solution for improving epidemiological surveillance of DENV in low resource settings across Indonesia, with the potential to facilitate a better understanding of transmission dynamics within the country. This portable and relatively affordable method for DENV sequencing was capable of generating near-complete coding-region coverage, even from low concentration samples and the resulting consensus sequences were sufficient for precise placement by phylogenetic analysis with strong support, demonstrating the method’s potential for generating data for future epidemiological studies, such as that performed in South America [27]. The accuracies of the Nanopore-generated consensus sequences here were comparable to those reported by previous Nanopore studies [26]. However, the remaining inaccuracies in Nanopore-generated sequences mean that less error-prone sequencing platforms will continue to be preferable when speed of sequencing is not paramount or for studies where the highest precision is required.

## Supplementary information

**Additional file 1.** Multiplex PCR primer information. List of PCR primer sequences used for multiplex amplification of dengue virus serotypes 1–4. Primer positions indicate the expected binding location based on the RefSeq genome for each serotype, with the appropriate accession number indicated in brackets. Two multiplex PCR reactions should be performed per sample, using either primers indicated as pool 1 or pool 2 in separate reactions.

## Data Availability

The datasets supporting the conclusions of this article are available in the ENA database BioProject number PRJEB35896.

## Abbreviations

DENV: Dengue virus
ONT: Oxford Nanopore Technologies
